# Association of chemerin levels and bone mineral density in Chinese obese postmenopausal women

**DOI:** 10.1097/MD.0000000000004583

**Published:** 2016-09-02

**Authors:** Liang Shi, Chaoming Mao, Xuefeng Wang, Rencong Liu, Lin Li, Xiao Mou, Ping Xu, Hongli Li, Chengcheng Xu, Guoyue Yuan, Bin Wang, Hao Zhang

**Affiliations:** a Department of Nuclear Medicine, Affiliated Hospital of Jiangsu University; b Department of Nuclear Medicine, Nanjing First Hospital; c Department of Endocrine and Metabolic Diseases, Affiliated Hospital of Jiangsu University; d Department of Laboratory Medicine, Nantong Tumor Hospital; e Department of Endocrine and Metabolic Diseases, Affiliated Hospital of Jiangsu University; f Department of ICU, Affiliated Hospital of Jiangsu University; g Emergency Medicine Center, Affiliated Hospital of Jiangsu University, China.

**Keywords:** bone mineral density, chemerin, cytokines, obese, osteoporosis

## Abstract

Increasing evidence suggests the association between obesity and bone metabolism. However, whether excessive fat accumulation has a beneficial or adverse effect on bone health remains controversial. Chemerin is a novel adipocyte-derived hormone and a chemoattractant cytokine that regulates adipogenesis. This study was performed to investigate the associations of serum chemerin with bone mineral density (BMD) and serum pro-inflammatory cytokine levels in 543 Chinese obese postmenopausal women. BMD of the femoral neck and lumbar spine, lean mass, and fat mass were measured using dual energy X-ray absorptiometry. Anthropometric assessment and laboratory measurements were performed. The age, time after menopause, and fat mass were negatively correlated with femoral and lumbar BMD, whereas lean mass was positively correlated with aforementioned variables. Furthermore, BMD at the lumbar spine was inversely associated with serum chemerin and TNF-α levels (*r* = −0.155, *P* = 0.001; *r* = −0.147, *P* = 0.001). Multiple linear regression analyses showed that serum chemerin levels were negatively correlated with BMD at the lumbar site after controlling for the age, lean, and fat mass (*β* = −0.125, *P* = 0.001). Chronic low-grade inflammation state in obese population has an inverse effect on bone mass. Chemerin as an adipocytokine and chemoattractant negatively affects the bone mass of Chinese obese postmenopausal women. Further studies are needed to confirm the potential role of chemerin in the crosstalk between bone and fat accumulation in obese population.

## 1 Introduction

Obesity is positively associated with bone mass.^[1]^ Many reports have supported the protective effect of obesity on bone mineral density (BMD) because mechanical loading conferred by body weight promotes bone formation.^[2]^ However, adipose tissue, as the largest endocrine organ in the human body, functions differently in the bone.^[3]^ Adipose tissue-associated hormone and adipose-modulated biochemical factors, such as estrogen^[4]^ and adipokines, may influence the relationship between fat and bone.^[5,6]^

Obesity is strongly associated with chronic, low-grade, and systemic inflammation state. Inflammatory components have also been shown to significantly affect the bone. Increased circulating and tissue pro-inflammatory cytokines in obesity are involved in bone metabolism through multiple pathways.^[1,7]^ Tumor necrosis factor-α (TNF-α) and interleukin-6 (IL-6) may promote osteoclast activity and bone resorption by modifying the receptor activator of NF-κB (RANK)/RANK ligand/osteoprotegerin pathway.^[8,9]^

Chemerin, an 18 kDa protein chemoattractant, is secreted by adipocytes mainly derived from fat^[10]^ and other tissues.^[11–13]^ This compound is known as a novel adipocytokine. Chemerin may be at the crossroads of inflammation and obesity because of its dual role in immune system and metabolism.^[14]^ Serum chemerin concentrations are increased in overweight and obese subjects.^[15–17]^ Moreover, chemerin levels are positively correlated with different obesity measures, such as BMI, and well-established markers of inflammation.^[10,18,19]^ In vitro studies have shown that chemerin regulates adipocyte development and metabolic function.^[20,21]^

Recent studies, however, have shown that chemerin may have a role in regulating bone metabolism. Expression and secretion of chemerin increased dramatically with adipocyte differentiation of preosteoblast and primary bone marrow stromal cells (BMSCs). Chemerin/chemokine-like receptor 1 (CMKLR1) signaling pathway may play an important role in regulating adipogenesis and osteoblastogenesis of bone marrow-derived precursor cells.^[22]^ Peroxisome proliferator-activated receptor gamma (PPARγ) significantly induced chemerin expression and secretion in BMSCs.^[23]^ Chemerin and its receptor CMKLR1 are expressed in hematopoietic stem cell (HSC) and secreted into the extracellular media. In addition, chemerin promotes osteoclastogenesis.^[24]^

The relationship between chemerin and BMD has not been fully elucidated, although the potential effect of adipocytokine chemerin on bone metabolism has been suggested. We measured serum chemerin concentrations, BMD and pro-inflammatory cytokines in human obese population to investigate the possible role of chemerin in the relationship between obesity and bone metabolism. We recruited postmenopausal women to exclude possible confounding factors.

## 2 Materials and methods

### 2.1 Subjects

A total of 543 obese (BMI ≥30 kg/m^2^) postmenopausal women from health examination centers of the Affiliated Hospital of Jiangsu University and Nanjing First Hospital were recruited into the study from September 2011 to April 2015. The age was 62.88 ± 6.74 years (range, 50–77). All participants had to be in menopause for at least 12 months. Information on the medical history of the study population was obtained using a detailed questionnaire and physical examination. Exclusion criteria were as follows: secondary causes of osteoporosis (rheumatoid arthritis, thyroid and parathyroid disorders, Cushing disease, malabsorption, osteomalacia, steroid-induced osteoporosis, immobilization, malignancy, renal osteodystrophy, liver diseases, other metabolic diseases except obesity, addiction to smoking, or alcohol); and drug addiction or use of medication known to influence bone metabolism, adipocytokines, and pro-inflammatory cytokines levels (calcium, vitamin D, corticosteroids, calcitonin, bisphosphonates, anti-vitamin K agents, selective estrogen-modulating agents, diuretics, β-blockers, heparin, antiepileptic drugs, statins, thiarizonaolidinediones, and regular or frequent use of non-steroidal anti-inflammatory drugs). Written informed consents were obtained from all subjects prior to inclusion into the study. The study was conducted according to the principles outlined in the Declaration of Helsinki and was approved by the Medical Ethics Committee of Jiangsu University (Zhenjiang, China).

### 2.2 Anthropometric assessment

Body weight and height were recorded in light clothing and no shoes. Measurements were taken to the nearest 0.1 kg and 0.1 cm, respectively. BMI was calculated (body weight/height^2^). Waist circumference (WC) was measured midway between the costal margins and iliac crest. Hip circumference was defined at the level of the greater trochanters. Body composition (total body lean and fat masses), left hip, and lumbar spine (L1–L4) BMD (kg/m^2^) were measured by dual energy X-ray absorptiometry machine (Lumar Prodigy Advance, GE. Healthcare, Madison, WI) according to standard protocol. All subjects were tested by the same operator during the study to eliminate operator discrepancies. A total of 35 participants agreed to undergo three scans on the same day to calculate the precision. Coefficients of variation (precision) of the measurements were 0.9% for body composition, 0.8% for left hip BMD, and 1.0% for lumbar spine BMD. These anthropometric results were assessed in a fasting morning and measured in duplicate by the same operator.

### 2.3 Laboratory measurements

A fasting serum sample was taken on the same day of anthropometric assessment and stored at −80 °C prior to analysis. Analyses were performed at the Nuclear Medicine Research Center. Chemerin was measured using enzyme-linked immune sorbent assay (ELISA) (Uscn Life Science Inc., Wuhan, China) with a detection limit of 0.1 ng/mL. IL-6 was analyzed using an ELISA commercially available kit (R&D Systems, Inc., Minneapolis, MN) with a detection limit of 0.7 pg/mL. Serum TNF-α concentrations were measured using an ELISA commercially kit (R&D Systems, Inc., Minneapolis, MN). The detection limit of the assay was 0.191 pg/mL.

### 2.4 Statistical analysis

Descriptive data were presented as mean values with standard deviation (SD) or median with interquartile range. Normal distribution of data was evaluated by Kolmogorov–Smirnov test. Logarithmic transformations were performed for statistical analysis because the distribution of chemerin was skewed.

Pearson correlation analysis was used to evaluate the relationships between chemerin values and the anthropometric and pro-inflammatory cytokines. Partial correlation analysis was performed to assess the association with adjustment for age, lean mass, and fat mass. Standard multiple linear regression models were used to assess the association between circulation chemerin levels (independent variables) and BMDs at different sites (dependent variables). The models were adjusted for age, lean mass, and fat mass. *P* values <0.05 was considered statistically significant. Statistical analyses were performed using SPSS 17.0 (SPSS, Inc., Chicago, IL).

Using the NCSS/PASS 2000 program, a sample size power calculation indicated that 543 participants were sufficient to perform the study with a power of 99% and an alpha error of 5%.

## 3 Results

### 3.1 Descriptive statistics

The general characteristics, anthropometric assessment, chemerin, and pro-inflammatory cytokine results are shown in Table 1. Information from 543 Chinese obese postmenopausal women, with ages ranging from 50 to 77 years, is provided in Table 1. The time after menopause (mean ± SD) of the women was 13.04 ± 7.01 years. According to the World Health Organization criteria, 97 women (17.9%) were considered osteoporotic.

**Table 1:**
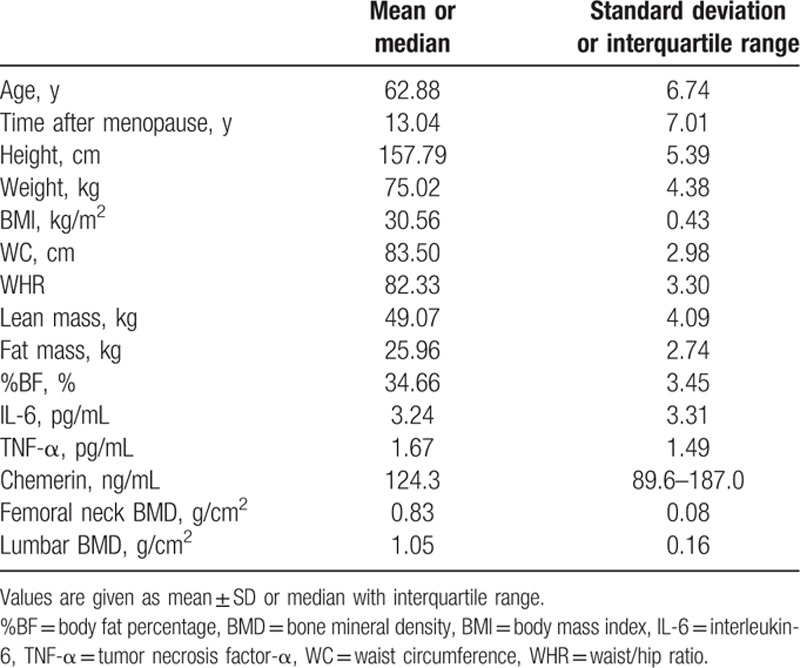
General characteristics of obese Chinese postmenopausal population (543 subjects).

### 3.2 Association of chemerin with age and obese anthropometric parameter

Table 2 shows unadjusted and adjusted correlations between chemerin and obese anthropometric parameters. Serum chemerin levels showed positive correlations with age, fat mass, %BF, and WC. Correlations between serum chemerin levels and fat mass (*r* = 0.107, *P* = 0.012) and %BF (*r* = 0.130, *P* = 0.002) persisted after adjustment for the age. Correlation between serum chemerin levels and WC (*r* = 0.130, *P* = 0.002) was even after adjustment for age and fat mass.

**Table 2:**
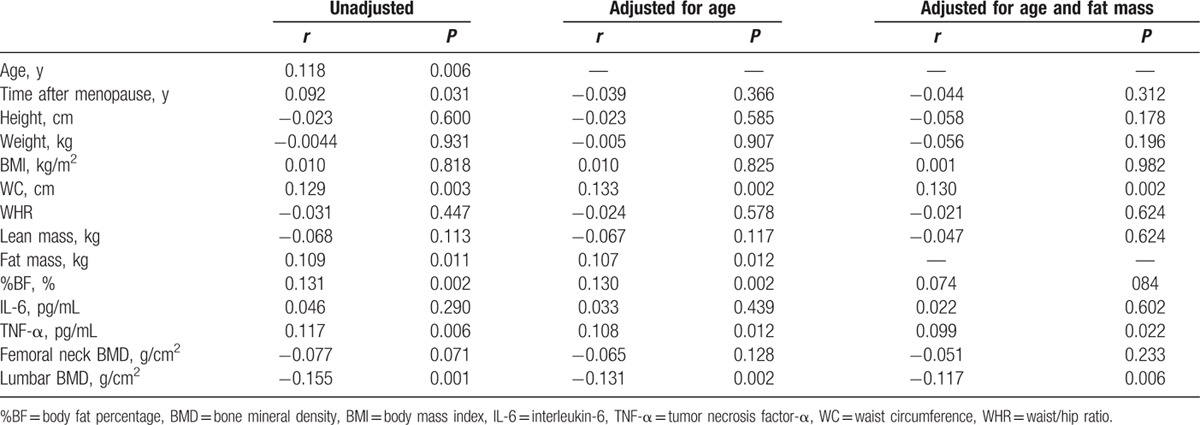
The correlation between chemerin and bone-related parameters in obese Chinese postmenopausal women.

### 3.3 Association of chemerin and pro-inflammatory cytokines

Chemerin showed a positive correlation with TNF-α after adjustment for age and fat mass (*r* = 0.099, *P* = 0.022, Table 2). However, no significant correlation was found between chemerin and IL-6 in postmenopausal women.

### 3.4 Association of chemerin and BMD

Bivariate correlation analysis showed negative correlations between chemerin and BMD at the lumbar spine (L1–L4) (*r* = −0.155, *P* = 0.001, Table 2). The correlation remained significant (*r* = −0.131, *P* = 0.002 and *r* = −0.117, *P* = 0.006) even after adjustment for age and fat mass. No significant correlation was found between chemerin and BMD at the femur neck in postmenopausal women.

Table 3 shows the results of multiple linear regression analyses for the correlation between circulating chemerin levels and BMD at both sites after controlling for age, lean mass, and fat mass.

**Table 3:**
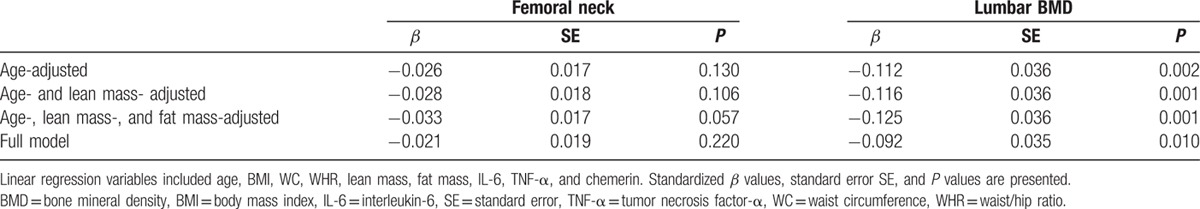
Multiple linear regression analysis for the association between chemerin (independent variables) and bone mineral density (dependent variables) in obese postmenopausal women.

In multiple regression analyses, serum chemerin levels showed significant negative correlation with BMD at the lumbar site after controlling for the age, lean mass, and fat mass (*β* = −0.125, *P* = 0.001).

### 3.5 Association of body composition, pro-inflammatory cytokines, and BMD

BMD measurements of the femur neck and lumbar spine were inversely correlated with age, time after menopause, and fat mass but positively correlated with lean mass (Table 3). The relationship of TNF-α to BMD was inverse in the lumbar spine (*r* = −0.147, *P* = 0.001), whereas no significant correlation was found in the femur neck. However, no significant correlations were found between IL-6 and BMD at both sites in postmenopausal women (Table 4).

**Table 4:**
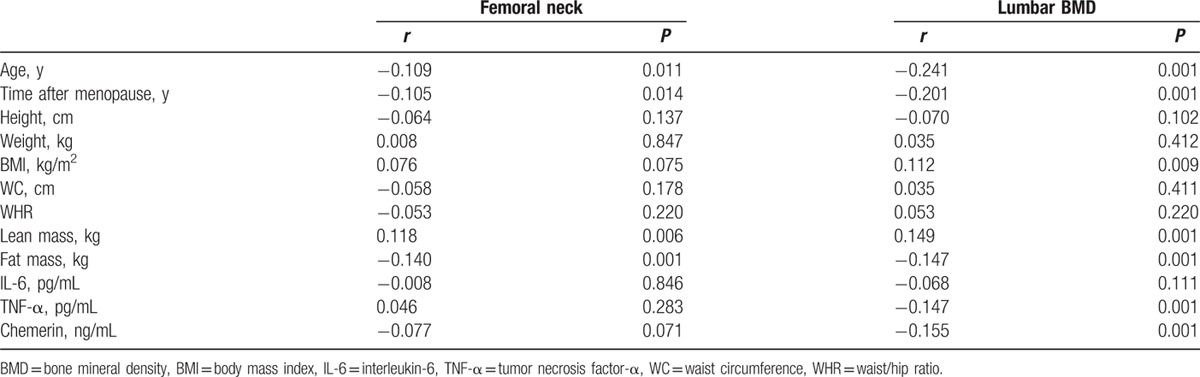
Correlation between bone-related parameters and BMD in obese Chinese postmenopausal women.

## 4 Discussion

Chemerin, which is released by adipose tissue, is strongly correlated with obesity.^[10]^ The relationship between serum concentrations of chemerin and BMD has not been extensively investigated despite the close association of obesity and BMD.^[1]^ In the current study, we found that serum chemerin level was positively associated with TNF-α and negatively associated with BMD of the lumbar spine. Moreover, chemerin served as an independent negative predictor of BMD. These findings support the hypothesis that chemerin may play a significant role in regulating bone mass in obesity.

Chemerin has shown a strong association with BMD. Several characteristics of chemerin provide plausible pathways for modulation of bone metabolism. Chemerin is widely expressed by various cell types such asadipocytes, osteoblasts, HSC, mesenchymal stem cells (MSCs), and macrophages.^[21–24]^ The multipotent role of MSCs has been proposed as a key mediator in the pathogenesis of osteoporosis, and the reduction in bone mass is partly believed to result from common precursor cells that become fat instead of bone.^[25]^ Chemerin is a negative regulator during bone formation.^[3]^ Chemerin stimulates adipocyte differentiation of BMSCs by activation of CMKLR1. Knockdown of chemerin gene increased osteoblast marker gene expression and mineralization after osteoblastogenic stimulation.^[22]^ In vivo, rosiglitazone elevated chemerin mRNA levels in adipose tissue and bone marrow along with an increase in circulating chemerin levels in mice.^[23]^ Chemerin regulated osteoclast differentiation of HSCs by modulating intracellular calcium and NFAT2 expression/activation.^[24]^ These results confirm that chemerin may negatively affect bone metabolism by inhibiting bone formation in the bone marrow.

Some studies have shown that PPARγ agonist rosiglitazone increased marrow fat, deteriorated trabecular architecture, and diminished bone density.^[26,27]^ PPARγ induced chemerin expression and partially rescued the loss of adipogenesis caused by knockdown of chemerin.^[22]^ Overexpression of PPARγ in obese peripheral blood mononuclear cells (PBMCs) of an obese individual may have a critical role in the relationship between obesity and bone loss.^[28]^ Thus, PPARγ agonist-induced bone mass loss maybe partially and indirectly caused by increased chemerin level which would stimulate more fat than osteoblasts in bone marrow and decrease bone formation.

Chemerin, which is newly identified as an adipokine in human adipose tissue, modulates adipogenesis and adipocyte metabolism by its paracrine or endocrine functions.^[20,21]^ Chemerin and chemerinR mRNA expression were dramatically upregulated during the differentiation from human preadipocytes into adipocytes.^[20]^ Chemerin levels were positively correlated with measures of obesity and metabolic syndrome.^[10,17,29]^ Chemerin also served as a significant predictor of metabolically healthy obesity.^[30]^ Obesity is associated with low-grade inflammation.^[31]^ Chemerin, as a chemo-attractant protein, also has a complex role in inflammation.^[32]^ Increased levels of chemerin in obesity were also related to inflammation.^[19]^ We showed that chemerin was significantly positively correlated with TNF-α in obese population, which is consistent with the result from a previous study.^[33]^ PBMC is proposed as the representative of the inflammatory status in obesity, and PBMC gene expression levels of chemerin are strongly upregulated in obesity.^[34]^ These phenomena indicated that chemerin may contribute to the low-grade chronic inflammation that characterizes obesity. Chemerin activated the intracellular signaling cascades MAPKs and Akt, followed by an enhanced secretion of pro-inflammatory cytokines IL-6 and TNF-α, incultured human articular chondrocytes.^[35]^ TNF-α treatment of 3T3-L1 adipocytes increases bioactive chemerin levels and may enhance prochemerin synthesis and secretion from adipocytes.^[36]^ Anti-TNF therapy reduces serum levels of chemerin in rheumatoid arthritis, which is associated with the decrease in serum levels of IL-6 and macrophage migration inhibitory factor.^[37]^ Dual role of chemerin in metabolism and inflammation may provide a link between chronic obesity and inflammation.^[14]^

Chronic low-grade systemic inflammatory state has been associated with osseous metabolism.^[38]^ Chronic inflammation and increased pro-inflammatory cytokines TNF-α and IL-6 induce bone resorption and bone loss in a number of diseases, such as periodontitis,^[39]^ rheumatoid arthritis,^[40]^ and inflammatory bowel disease.^[41]^ These pro-inflammatory cytokines in obesity stimulates osteoclastactivity and bone resorption through RANKL/RANK/OPG pathways.^[7–9]^ In this study, serum levels of chemerin, as well as TNF-α, were negatively associated with BMD measurements of the lumbar spine in obese population, which is in line with the study by He et al,^[42]^ who also found negative effect of chemerin on BMD in osteoporotic patients. Moreover, our results showed that chemerin levels were independently associated with BMD at the lumbar spine site. Thus, results show a relationship of obesity–chemerin–pro-inflammatory cytokines–osteoporosis, that is, fat-derived chemerin and pro-inflammatory cytokines crosstalked with each other. This process exerts a complex effect on bone loss in obese postmenopausal women.

We found a negative association between fat mass and BMD at the lumbar spine in obese postmenopausal women. Positive association between body weight and BMD was previously demonstrated in many populations.^[43]^ However, the specific role of lean and fat masses, which constitute the body weight, in BMD remains controversial.^[44,45]^ Some studies showed that fat mass maybe a negative independent predictor of BMD,^[46]^ and lean mass has a strong positive influence on BMD regardless of age or sex.^[47]^ Meta-analysis revealed that exercise exerted a significant positive effect on BMD with increased lean body mass in postmenopausal women^[48]^ and in men.^[49]^ At the same time, exercise induced fat mass loss with reduced serum chemerin and improved systemic inflammation in obese men.^[50]^ These studies may indicate that excessive body fat accumulation accompanied by elevated chemerin and related chronic inflammation state may in turn had a negative effect on BMD. Large-scale studies, both in vivo and in vitro, are warranted to evaluate the relation among chemerin, obesity, and osteoporosis.

Some limitations of our study should be noted. First, the cross-sectional nature of this study limited the interpretation of our results, and no inferences of causality could be formulated. The hypothesis we made to attempt to explain the relationship between chemerin and BMD needs further and more detailed investigations. Second, the study included only Chinese obese postmenopausal women. Thus, the results may not be generalized to other populations, such as women in other ethnic groups and ages or men and adolescents. Third, we were unable to directly measure sex hormone, namely, estrogens and androstendione, which changes with age and duration of postmenopausal and affects fat redistribution and bone mass. Fourth, bone density scan (DXA) measurements are influenced by body position, body size, and bone size.^[51,52]^ Bias from the difference of areal BMD (by DXA) from the real BMD will affect the relationship between chemerin and bone mass. Fifth, given that chemerin is involved in bone, fat, and inflammation, we did not measure other adipocytokines, inflammatory markers besides TNF-α and IL-6, and bone metabolic markers. Lastly, the data of the lifestyle of participants, such as exercise, which may affect both circulation chemerin levels and BMD were not evaluated.

The relationship between obesity and BMD in obese population is clinically important because excessive body fat might induce local and systemic low-grade inflammation, causing a risk factor for bone loss. Any therapeutic interventions for obesity that modify body fat metabolism may affect BMD. We speculated that chemerin, known as an adipokine and chemoattractant, might provide a link among fat, inflammation, and bone. Few studies have attempted to investigate the effects of fat and chemerin on BMD in obese population. Therefore, in this study we investigated the relationship between chemerin, BMD, and pro-inflammatory cytokines in obese population, aiming to determine whether adipokine chemerin and systemic low-grade inflammation are associated with BMD in obese postmenopausal women.

In conclusion, this is the first report that identified unique associations between BMD, fat mass, inflammation, and chemerin. Chemerin is a novel adipocyte-derived hormone previously implicated in obesity. This compound is also a chemokine and plays a role in systemic immune diseases. Our findings show that serum chemerin levels were positively associated with pro-inflammatory cytokines and negatively associated with BMD of the lumbar spine in Chinese obese postmenopausal women. Chemerin apparently plays a negative role in the relationship between fat mass and BMD. Further prospective investigations in different population groups are necessary to elucidate these associations. Chemerin is a promising biomarker and target for the prevention and treatment of osteoporosis.
