# Association of chemerin levels and bone mineral density in Chinese obese postmenopausal women: Erratum

**DOI:** 10.1097/01.md.0000508235.21956.1e

**Published:** 2016-10-28

In the article “Association of chemerin levels and bone mineral density in Chinese obese postmenopausal women”,^[1]^ which appeared in Volume 95, Issue 35 of *Medicine*, Dr. Bing Wan's name was originally misspelled. The article has since been corrected online.
